# *Saxifraga stolonifera* curtis for ear inflammation: antibacterial efficacy and key bioactive constituents

**DOI:** 10.1186/s12906-026-05346-x

**Published:** 2026-04-13

**Authors:** Fukui Shen, Hongyu Liu, Wen Yang, Tian-Tian Tong, Shiyu Guo, Zhihao Wei, Wenping Zhang, Jihui Fan, Yuqing Zhang, Hao Huang

**Affiliations:** 1 Jiangxi Province Key Laboratory of Pharmacology of Traditional Chinese Medicine, School of Pharmacy, Gannan Medical University, Ganzhou, 341000 China; 2 State Key Laboratory of Medicinal Chemical Biology, College of Pharmacy and Tianjin Key Laboratory of Molecular Drug Research, Nankai University, Tianjin, 300353 China; 3 State Key Laboratory of Quality Research in Chinese Medicine, Macau University of Science and Technology, Avenida Wai Long, Taipa, Macau SAR China

**Keywords:** *Saxifraga stolonifera*, Otitis media, Antibacterial, Anti-inflammatory, UHPLC-Q-TOF

## Abstract

**Background:**

*Saxifraga stolonifera* Curtis (SSC), a highly esteemed Traditional Chinese Medicine with profound ethnomedicinal value, has been historically employed to treat inflammatory ear disorders. Contemporary preclinical studies have verified its effectiveness in combating otitis media (OM). However, the underlying pharmacological mechanisms, particularly its anti-inflammatory and antibacterial properties remain unclear.

**Purpose:**

This study aimed to systematically elucidate the chemical basis, antibacterial activity and molecular mechanisms underlying SSC’s anti-OM activity.

**Methods:**

UHPLC-Q-TOF-MS was employed for comprehensive phytochemical profiling of SSC total extracts. Antibacterial assays were conducted against three OM-associated bacterial strains: *Staphylococcus aureus* (SA), *Pseudomonas aeruginosa* (PA), and *Klebsiella pneumoniae* (KP), using SSC total extracts and its total flavonoid (TF). Network pharmacology analysis identified OM-related targets and pathways, followed by molecular docking validation. qPCR was used to assess mRNA expression of inflammatory cytokines (*IL-6*,* TNF-α*,* IL-1*β) in LPS-induced J774A.1 macrophages.

**Results:**

Twenty-five bioactive compounds were characterized, including 11 newly reported constituents. Antibacterial results showed SSC exhibited the strongest inhibitory effect against KP, while TF showed relatively strong activity against PA; overall, SSC outperformed TF, suggesting contributions from non-flavonoid components. Network pharmacology revealed 11 core targets interconnected with inflammatory pathways. Molecular docking confirmed strong binding affinities between key components (bergenin, 11-O-galloylbergenin) and targets (HSP90AA1, EGFR). SSC total extracts significantly downregulated pro-inflammatory cytokine mRNA levels in vitro.

**Conclusion:**

SSC exerts anti-OM effects through a “dual mechanism”: antibacterial activity (with SSC total extract showing stronger efficacy than TF) and anti-inflammatory regulation *via* TNF/MAPK/NF-κB pathways. This integrative study validates SSC’s traditional use and provides a basis for developing SSC-based therapeutics for OM.

**Graphical Abstract:**

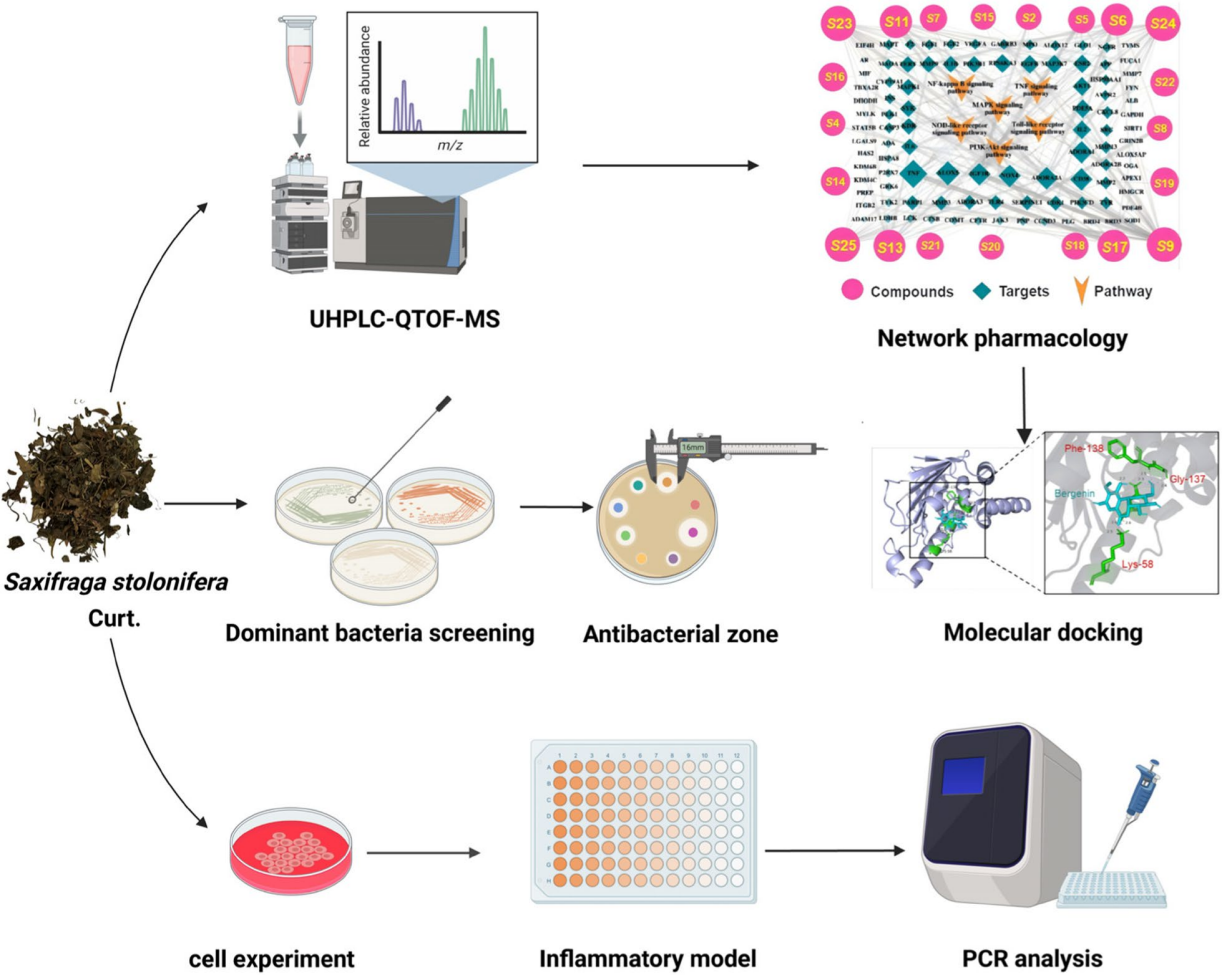

**Supplementary Information:**

The online version contains supplementary material available at 10.1186/s12906-026-05346-x.

## Introduction

Otitis media (OM) is a highly prevalent pediatric ear disease, affecting approximately 80% of American children; untreated cases can lead to hearing impairment or permanent hearing loss [1, 2]. Current conventional treatments, including antibiotics, glucocorticoids, and surgery, pose significant challenges such as antibiotic resistance, disease recurrence and adverse effects [3, 4]. Notably, bacterial infection is a key driver of OM progression: clinically relevant strains, such as *Staphylococcus aureus* (SA, Gram-positive), *Pseudomonas aeruginosa* (PA, Gram-negative), and *Klebsiella pneumoniae* (KP, Gram-negative), are frequently isolated from OM patients, which exacerbates inflammatory responses and delays recovery [5]. Thus, there is an urgent need for therapeutic agents with dual anti-inflammatory and antibacterial activities for OM management.

As a traditional herbal medicine, *Saxifraga stolonifera* Curtis (SSC) has been shown to be rich in various chemical constituents and possesses significant properties of promoting hypo-lipidemia [6], managing ischemic heart disease [7] and facilitating detoxification [8]. Modern pharmacological studies reveal that SSC exhibits extensive biological activities, including anti-inflammatory, antibacterial, antitumor, antitussive, and hepatoprotective effects [9–11]. Wang et al. found that the bergenin as the main components of SSC total extracts improves experimental colitis in mice by inhibiting macrophage activation through the PPARγ/SIRT1/NF-κB-p65 pathway, providing evidence for the potential of bergenin as an anti-ulcerative colitis drug [12]. Kawahara et al. discovered a novel physiological function of SSC, which is the suppression of TLR2 expression and TLR2-mediated inflammation in human skin keratinocytes, offering significant insights into its anti-inflammatory effects [13]. Recently the potential antiviral activity of SSC has also been reported and raised concerns. As recorded in the “Compendium of Materia Medica,” SSC has been used for the treatment of otitis media for centuries, and modern pharmacological studies confirm its strong anti-inflammatory activity, effectively suppressing both acute and chronic inflammation [14]. A small-scale clinical demonstrated that topical application of SSC for 7 days resulted in reduced secretions and pain relief in 78% of 45 patients with OM, with no adverse reactions observed [15]. However, the anti-inflammatory and antibacterial mechanisms underlying SSC’s therapeutic effect on OM remain unsystematically investigated.

SSC historically used to treat ear disorders, emerges as a promising candidate in this context. This potential is further supported by recent studies: herbal extract-based ear drops have shown enhanced efficacy in alleviating OM symptoms [16, 17]. Previous studies on SSC have focused on its anti-inflammatory effects and flavonoid-rich fractions, with flavonoids widely regarded as the primary bioactive components for antibacterial activity in plants [18]. However, no study has systematically evaluated SSC’s antibacterial activity against OM-associated bacteria, nor compared the efficacy of SSC total extract versus its total flavonoid extract, a gap that limits understanding of SSC’s full therapeutic potential for OM.

To address this, we integrated multiple approaches: (1) UHPLC-Q-TOF-MS for comprehensive phytochemical profiling of SSC; (2) antibacterial assays to assess SSC and TF against SA, PA, and KP, and identify differences in their efficacy; (3) network pharmacology to predict OM-related targets and pathways; (4) molecular docking to validate component-target binding; and (5) qPCR to verify anti-inflammatory activity in LPS-induced macrophages. This study aims to clarify SSC’s “anti-inflammatory + antibacterial” dual mechanisms against OM, and highlight the potential of non-flavonoid components to contribute to its therapeutic effects, providing a scientific basis for SSC-based OM therapeutics.

## Materials and methods

### Materials

SSC (batch No.20200705, collected from Chengdu City, China, harvest time in July, whole plant) was purchased from Bozhou Medicinal Material Market (Anhui, China). Reagents used included chromatographic grade methanol and acetonitrile (ACN, Sinopharm Chemical Reagent Co., Ltd.), chromatographic grade formic acid (FA, Aladdin Reagent Shanghai Co., Ltd.), chromatographic water (Watsons purified water, Watsons Co., Ltd.), high-glucose DMEM medium (Wuhan Procell Life Science & Technology Co., Ltd.), fetal bovine serum (Biological Industries), penicillin-streptomycin mixture (100×), PBS (both from Beijing Solarbio Science & Technology Co., Ltd.), *Staphylococcus aureus* (Gram-positive, strain standard No.ATCC6538, Shanghai Luwei Biotechnology Co., Ltd.), *Pseudomonas aeruginosa* (Gram-negative, strain standard No.ATCC27853, Shanghai Luwei Biotechnology Co., Ltd.), *Klebsiella pneumoniae* (Gram-negative, strain standard No.ATCC13883, Shanghai Luwei Biotechnology Co., Ltd.), lipopolysaccharide (LPS, *Escherichia coli* 055:B5, Sigma-Aldrich), Cell Counting Kit-8 (CCK-8, Shanghai Hongye Biotechnology Co., Ltd.), and PerfectStart™ Green qPCR SuperMix, TransZol Up Plus RNA, EasyScript One-Step gDNA Removal and cDNA Synthesis SuperMix (all from TransGen Biotech Co., Ltd.) for molecular biology experiments. J774A.1 mouse macrophage cells were purchased from Wuhan Procell Life Science & Technology Co., Ltd. Instruments employed included an Agilent 1290 Ultra Performance Liquid Chromatography (UPLC) system, an Agilent 6545 Quadrupole/Time of Flight (Q-TOF) mass spectrometer (MS) (both from Agilent Technologies, USA), and a QuantStudio™ 7 Flex Real-Time PCR System (Thermo Fisher Scientific, USA).

### Preparation of total extract solution of SSC and total flavonoid

The extraction was performed by refluxing 5.0 g of powdered SSC with 100 mL of 50%-70% ethanol in a Soxhlet extractor until a colorless and transparent liquid was obtained. The resulting mixture was then filtered, and the filtrate was collected. Results showed 70% ethanol yielded the highest relative bergenin content (16.92%) in the extract, leading to its final selection (Fig. S1). After concentration, the extract was reconstituted in methanol and made up to a final volume of 100 mL. The test solution was prepared by filtering a portion of this stock solution through a 0.22 μm organic phase membrane filter. The test solution was rotary evaporated at 40 °C, followed by freeze-drying for 48 h to obtain lyophilized powder. The powder was weighed, and the yield of 70% ethanolic extract of SSC was calculated to be 20.44%, which was used for subsequent experiments.

Additionally, for the extraction of total flavonoids from SSC, the water extraction and alcohol precipitation method was used. The dried SSC herbs were processed into a fine powder (passed through a No. 4 sieve). An aliquot (15 g) of the powder was extracted with 150 mL of water (1:10, w/v) at 90 °C for 30 min and then filtered. The filtrate was concentrated by rotary evaporation. Following the addition of ethanol to a final concentration of 75%, the solution was precipitated at 4 °C for 48 h. The resulting supernatant was collected, concentrated again, and freeze-dried to yield the total flavonoid extract. The total flavonoid extract was then weighed, and the yield of TF was calculated to be 8.02%.

### SSC antimicrobial activity assay

For the antibacterial activity assay, three strains—*Staphylococcus aureus* (SA, Gram-positive), *Pseudomonas aeruginosa* (PA, Gram-negative), and *Klebsiella pneumoniae* (KP, Gram-negative)—were used, with all stored in 20% glycerol at -80 °C. Prior to testing, strains were activated on Tryptic Soy Agar (TSA), then a single activated colony was inoculated into 7 mL Tryptic Soy Broth (TSB) and shake-cultured at 37 °C for 10 h to reach the logarithmic phase; the bacterial suspension was first adjusted to 10⁸ CFU/mL, then further diluted to 5 × 10⁵ CFU/mL for plate inoculation. Sterilized TSA was melted, cooled to 50 °C, poured (15 mL per dish) to solidify, then 50 µL of the diluted bacterial suspension was spread on each plate and left to absorb for 15 min. 6 mm-diameter wells (≥ 24 mm apart) were punched, and 100 µL of test solutions (100/200 mg/mL *Saxifraga stolonifera* total extract or total flavonoid extract) were added, with 15 µg/mL ciprofloxacin (positive control) and 0.1% DMSO (negative control) set (triplicates for each). Plates were incubated at 37 °C for 24 h, after which the outer diameter of each inhibition zone (including well diameter) was measured twice perpendicularly with a vernier caliper, and the mean ± standard deviation was calculated from triplicates.

Indicator bacterial suspensions in the logarithmic phase were standardized to approximately 1 × 10⁸ CFU/mL and diluted with TSB to a final inoculum concentration of 5 × 10⁵ CFU/mL. For MIC determination, serially diluted sample solutions were mixed with an equal volume of bacterial suspension in 96-well plates, with growth controls and negative controls included. After static incubation at 37 °C for 24 h, the MIC was defined as the lowest sample concentration showing no visible bacterial growth to the naked eye. Subsequently, MBC was assessed *via* the agar plate method following MIC testing: 100 µL of bacterial suspension was aspirated from each well without visible growth in the MIC assay, evenly spread on fresh drug-free TSB agar plates, and incubated at 37 °C for 24 h. Colony counting was performed, and the MBC was defined as the concentration yielding fewer than 10 CFU (*n* = 3).

### UHPLC-Q-TOF analysis of SSC

The chromatographic separation was performed on an Acquity UPLC BEH C18 column (2.1 mm × 100 mm, 1.7 μm) maintained at 40 °C. The mobile phase consisted of (A) 0.1% formic acid in water and (B) 0.1% formic acid in acetonitrile, delivered at a flow rate of 0.3 mL/min. Gradient elution was programmed as follows: 0–8 min, 2–15% B; 8–20 min, 15–35% B; 20–24 min, 35–60% B; 24–32 min, 60–100% B; 32–35 min, 100% B. The column was re-equilibrated with 2% B for 8 min prior to each analysis. The injection volume was 5 µL.

### Mass spectrometry conditions

Mass spectrometric analysis was conducted using an electrospray ionization source (ESI) in positive ion mode. The drying gas (N₂) was heated to 200 °C and delivered at a flow rate of 11 L/min, with a nebulizer pressure of 40 psi. The sheath gas (N₂) temperature and flow rate were set at 300 °C and 12 L/min, respectively. The capillary, nozzle, fragmentor, and skimmer voltages were maintained at 4.0 kV, 200 V, 175 V, and 65 V, respectively. Spectra were acquired over a mass-to-charge ratio (m/z) range of 100–1700 at a scan rate of 2.0 spectra/s. Continuous calibration was performed using reference masses at m/z 121.0509 and 922.0098. For targeted MS/MS analysis, collision energies of 10, 20, 30, and 40 eV were applied.

### Identification, isolation and purification of chemical components

Data acquisition and processing were performed using Agilent MassHunter Qualitative Analysis software (version B.06.00). A local database of SSC natural products was constructed by reviewing the pertinent literature and consulting the online Dictionary of Natural Products. This database was imported into the MassHunter workstation. Compound identification was conducted by utilizing the “search by database” function. During this process, a mass error tolerance of 5 ppm was set for matching the theoretical and measured molecular weights of the parent ions. The tentative identification of the main chemical components in SSC was achieved by comparing the characteristic fragments of the product ions.

Isolation process: 500 g of SSC freeze-dried powder was mixed with 500 g of column chromatography silica gel (200 ~ 300 mesh) at a ratio of 1:1 (g/g) for sample mixing. The column was packed and loaded, and the SSC extract was separated by column chromatography with petroleum ether, petroleum ether: ethyl acetate (100:1→0:1), ethyl acetate: methanol (10:1→1:1), and methanol as the mobile phases in turn. The separation results were detected by thin-layer chromatography (TLC), and the fractions containing the same compounds were combined. The fractions obtained by column chromatography were further separated and purified by repeated silica gel column chromatography, repeated gel column chromatography, and reversed-phase column chromatography to obtain monomer compounds.

### Acquisition and screening of component-target

Use the PubChem database (https://pubchem.ncbi.nlm.nih.gov/) to search for the identified chemical components in SSC, obtain their structural formulas, and export them in SDF format. For components not included in the PubChem database, combine literature research and use Chemdraw16.0 software to draw the component structures and save them as SDF files. Import the component structure SDF data into the SwissTargetPrediction database (http://swisstargetprediction.ch/), predict the action targets of the identified components in SSC, organize the search results to obtain the active component-target database of SSC.

### Construction of the OM disease target database

To ensure the accuracy and completeness of the data, searches were conducted in the Genecard database (https://www.genecards.org/) and the OMIM database (https://www.omim.org/) using the keywords “Otitis media,” “Acute otitis media,” and “Chronic otitis media.” The results were aggregated, and duplicate genes were removed to form the OM disease target database.

### Drawing of venn diagrams and protein-protein interaction (PPI) maps

The target points of the active components of SSC were mapped against the disease targets of OM. The intersection of the two sets of targets was obtained, which are the related targets for OM treatment with SSC. The component targets of SSC and the disease targets of OM were imported into the Venny2.1.0 online diagramming tool (https://bioinfogp.cnb.csic.es/tools/venny/) to obtain the Venn diagram of targets related to OM treatment with SSC. The related targets for OM treatment with SSC were imported into the String database (https://string-db.org/), with the protein species set to “Homo sapiens” and the target association confidence set to “high confidence 0.700.” Isolated nodes were hidden, and the TSV data of the target interaction network relationship was downloaded. The data was imported into Cytoscape 3.8.2 software to draw the Protein-Protein Interaction (PPI) network, and data visualization was performed. The Maximal Clique Centrality (MCC) algorithm of the CytoHubba plugin was used to screen out important hub targets in the network.

### KEGG signal pathway enrichment analysis

KEGG signal pathway enrichment analysis was employed [19], with the database screening parameter *P* Value Cutoff set at 0.05. The KEGG pathway enrichment data was downloaded, and data visualization analysis was performed on the top 20 KEGG pathways.

### Construction of the active component-disease target-signal pathway network

The active components-targets of SSC from 4.5 were matched with the OM disease targets from 4.6. The active components and targets with matching results were retained, while data without matching results were deleted. Combined with the enriched key signal pathways from 4.8, the active component-disease target-signal pathway database was obtained. The database information was imported into Cytoscape 3.8.2 software for data visualization, predicting the network relationship of the components, disease targets, and signal pathways of SSC in treating OM. The Network Analyzer plugin built into Cytoscape was used to analyze the network’s topological parameters, and the core components and core targets for SSC in treating otitis media were screened based on the degree of connectivity.

### Molecular docking evaluation

Based on the PPI network of targets related to OM treatment with SSC from 4.7 and the active component-disease target-signal pathway network from 4.9, core components with a high degree of connectivity were molecularly docked with core targets and hub targets. The 3D crystal structures of the core and hub target proteins were searched in the RCSB PDB database (https://www.rcsb.org/), and the target protein PDB files were downloaded. Preprocessing such as solvent removal, hydrogen addition, and charge balancing was performed on the target proteins using PyMol software [20]. The 3D structures of the core components were searched in the ZINC database (https://zinc.docking.org/) and saved in Mol2 format. The core and hub target proteins and core components were imported into the AutoDockTools-1.5.6 database for molecular docking evaluation analysis [21]. The evaluation results were plotted as a matrix heatmap, and some docking results were visualized in the UCSF Chimera software.

### Cell culture

J774A.1 mouse macrophage cells (Cat. No. CL-0370) were purchased from Wuhan Procell Life Science & Technology Co., Ltd. (Wuhan, China) and used at passages 10–25 after receipt. J774A.1 cells were placed in high-glucose DMEM medium containing 10% (v/v) fetal bovine serum, 1% (v/v) streptomycin, and penicillin at 37 °C and 5% CO_2_. According to the method in the reference, J774A.1 cells were incubated in high-glucose DMEM medium containing 100 ng·mL-1 LPS for 1 h to obtain the J774A.1 macrophage inflammatory model.

### CCK-8 assay

J774A.1 cells were seeded in a 96-well plate (5000 cells/well) at 100 µL per well. After adherence, they were treated with serum-free medium containing 0, 6.25, 12.5, 25, 50, 100, 200 µg/mL of SSC total extract. Each group had 6 replicates. After 24 h of action, 10 µL of CCK-8 reagent was added to each well and incubated in a CO_2_ incubator for 2 h. The absorbance values at 450 nm were measured using a microplate reader, and cell viability was calculated [22].

### PCR analysis

J774A.1 cells were seeded in a 6-well plate (5 × 10^5^ cells/well) and allowed to adhere overnight. The experiment was set up in normal, model, and dosing groups. The normal group was first treated with serum-free high-glucose DMEM medium for 24 h, replaced with fresh serum-free medium, and treated for 1 h. The model group was first treated with serum-free high-glucose DMEM medium for 24 h, replaced with LPS-containing serum-free medium (100 ng/mL), and treated for 1 h. The dosing groups were treated with high-glucose DMEM serum-free medium containing 50 µg·mL-1 of SSC total extract for 24 h, replaced with LPS-containing serum-free medium (100 ng/mL), and treated for 1 h. Each group had 3 replicates. Total RNA was extracted using a high-purity RNA extraction kit, and 2 µg of total RNA was reverse transcribed and de-genomized into cDNA. Real-time PCR amplification of *IL-6*, *TNF-α*, and *IL-1β* was performed using a real-time PCR detection system and SYBR, with a 20 µL amplification system and a program set at 94 °C for 30 s, 1 cycle; 94 °C for 5 s, 60 °C for 30 s, 45 cycles. *GAPDH* was used as an internal reference gene, and the relative expression of genes was analyzed using the 2-^ΔΔCt^ method. The primer sequences are shown in Table S1.

### Statistical analysis

All data were derived from at least three independent biological replicates (*n* = 3) and presented as mean±standard deviation (SD). Data were analyzed and processed using GraphPad Prism 10.0 software. One-way analysis of variance (ANOVA) was used to compare differences among groups, followed by Tukey’s post-hoc test for multiple comparisons. *p* < 0.05 was considered statistically significant. Different lowercase letters (a, b, c, d, e, f, g, h) indicate significant differences as determined by Tukey’s test.

qRT-PCR data were analyzed using the 2 − ^ΔΔCt^ method. The cycle threshold (Ct) values of target genes were first normalized to those of the housekeeping gene GAPDH to calculate ΔCt values. Subsequently, the ΔCt values of experimental groups were compared with the mean ΔCt value of the control group to obtain ΔΔCt values. Finally, relative expression levels were calculated as 2 − ^ΔΔCt^, with the expression level of the control group set to 1.

## Results and discussion

### Antibacterial activity

**Fig. 1:**
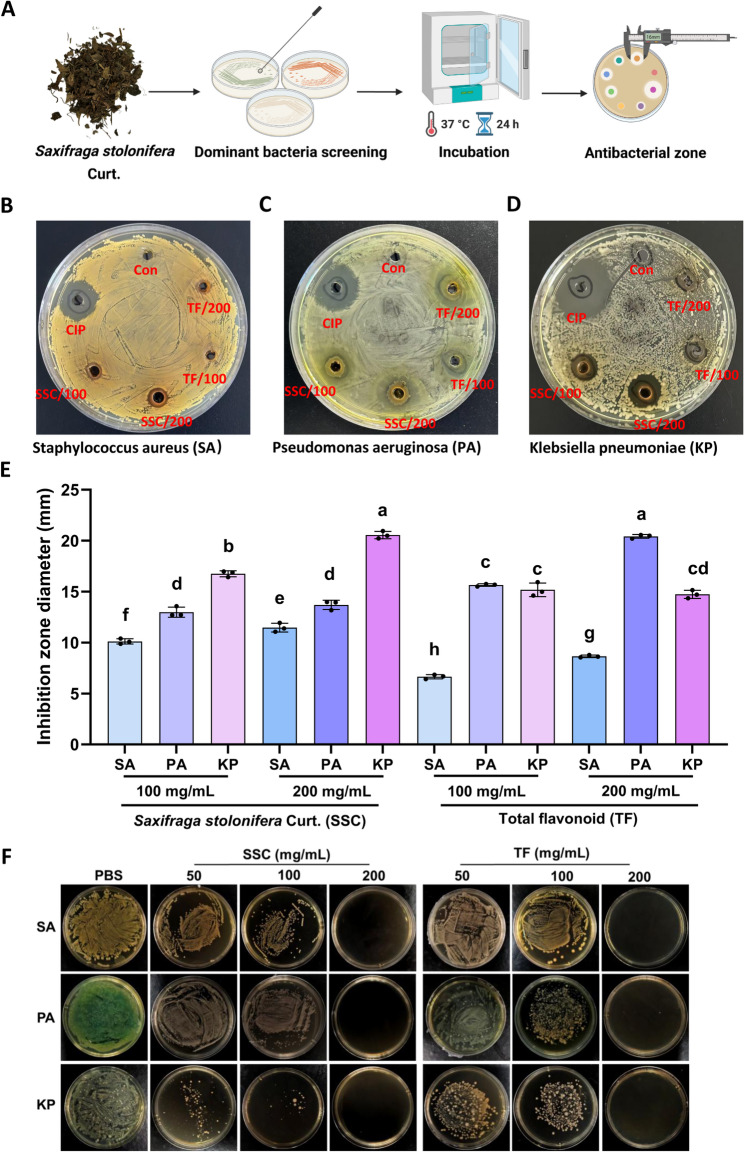
Visualization and analysis of antibacterial effects. **A** Schematic of the antibacterial assay workflow; **b**–**d** Representative agar plates showing inhibition zones of test samples against *SA*,* PA and KP*, CIP (ciprofloxacin, positive control), Con (0.1% DMSO, negative control);. **e **Quantitative analysis of inhibition zone diameters (mm, mean ± SD) for SSC and TF (100/200 mg/mL) against SA, PA, and KP (*n* = 3 biological replicates). Different lowercase letters (a, b, c, d, e, f, g, h) indicate significant differences as determined by Tukey’s test; **f **Determination of MBC of SSC and TF fraction against SA, PA and KP (*n* = 3 biological replicates)

To evaluate the antibacterial potential of *Saxifraga stolonifera* extract (SSC) and its total flavonoid extract (TF), antibacterial assays were conducted against three bacterial strains: *Staphylococcus aureus* (SA, Gram-positive), *Pseudomonas aeruginosa* (PA, Gram-negative), and *Klebsiella pneumoniae* (KP, Gram-negative). The experimental workflow is illustrated in Fig. 1A, while representative agar plates showing the inhibition zones for each strain are presented in Fig. 1B–D (CIP: ciprofloxacin, positive control; Con: 0.1% DMSO, negative control). As quantitated in Fig. 1E, the inhibition zone diameters (mean ± standard deviation, mm) against SA were 10.17 ± 0.251 (SSC at 100 mg/mL), 11.46 ± 0.441 (SSC at 200 mg/mL), 6.64 ± 0.22 (TF at 100 mg/mL), and 8.15 ± 0.119 (TF at 200 mg/mL). For PA, the corresponding values were 12.99 ± 0.497 (SSC/100), 13.69 ± 0.453 (SSC/200), 15.64 ± 0.126 (TF/100), and 20.42 ± 0.184 (TF/200). Against KP, the inhibition zone diameters measured 16.75 ± 0.291 (SSC/100), 20.55 ± 0.365 (SSC/200), 15.17 ± 0.67 (TF/100), and 14.73 ± 0.40 (TF/200). Results revealed that SSC exhibited the most potent inhibitory effect against KP, whereas TF showed relatively strong inhibitory activity against PA. Collectively, SSC demonstrated superior overall antibacterial efficacy compared to TF.

To validate the semi-quantitative agar well diffusion results, we supplemented minimum bactericidal concentration (MBC) assays, OD_600_-based minimum inhibitory concentration (MIC) determination was unfeasible due to the intense inherent color of SSC and TF (traditional Chinese medicine extracts), which would interfere with accurate readings. MBC results confirmed SSC’s antibacterial activity: 100 mg/mL against KP and 200 mg/mL against SA and PA. TF exhibited a uniform MBC of 200 mg/mL against all three pathogens (Fig. 1F). These quantitative data reinforce the reliability of the initial diffusion-based findings and clarify the bactericidal potency of SSC and its flavonoid fraction.

Notably, TF exhibited stronger inhibitory activity against PA than SSC (20.42 ± 0.184 mm vs. 13.69 ± 0.453 mm at 200 mg/mL), which may be attributed to specific flavonoids (e.g., quercetin, luteolin) in TF, previous studies have shown that quercetin can disrupt the outer membrane of Gram-negative bacteria like PA by binding to lipopolysaccharide [23]. In contrast, SSC’s superior activity against KP and SA suggests non-flavonoid components (e.g., bergenin) play a dominant role in inhibiting these strains. To fully characterize the bioactive components underlying the observed antibacterial effects, subsequent studies will employ liquid chromatography-mass spectrometry (LC-MS) to identify and quantify potential non-flavonoid antibacterial substances in SSC.

### Chemical composition analysis of SSC

**Fig. 2:**
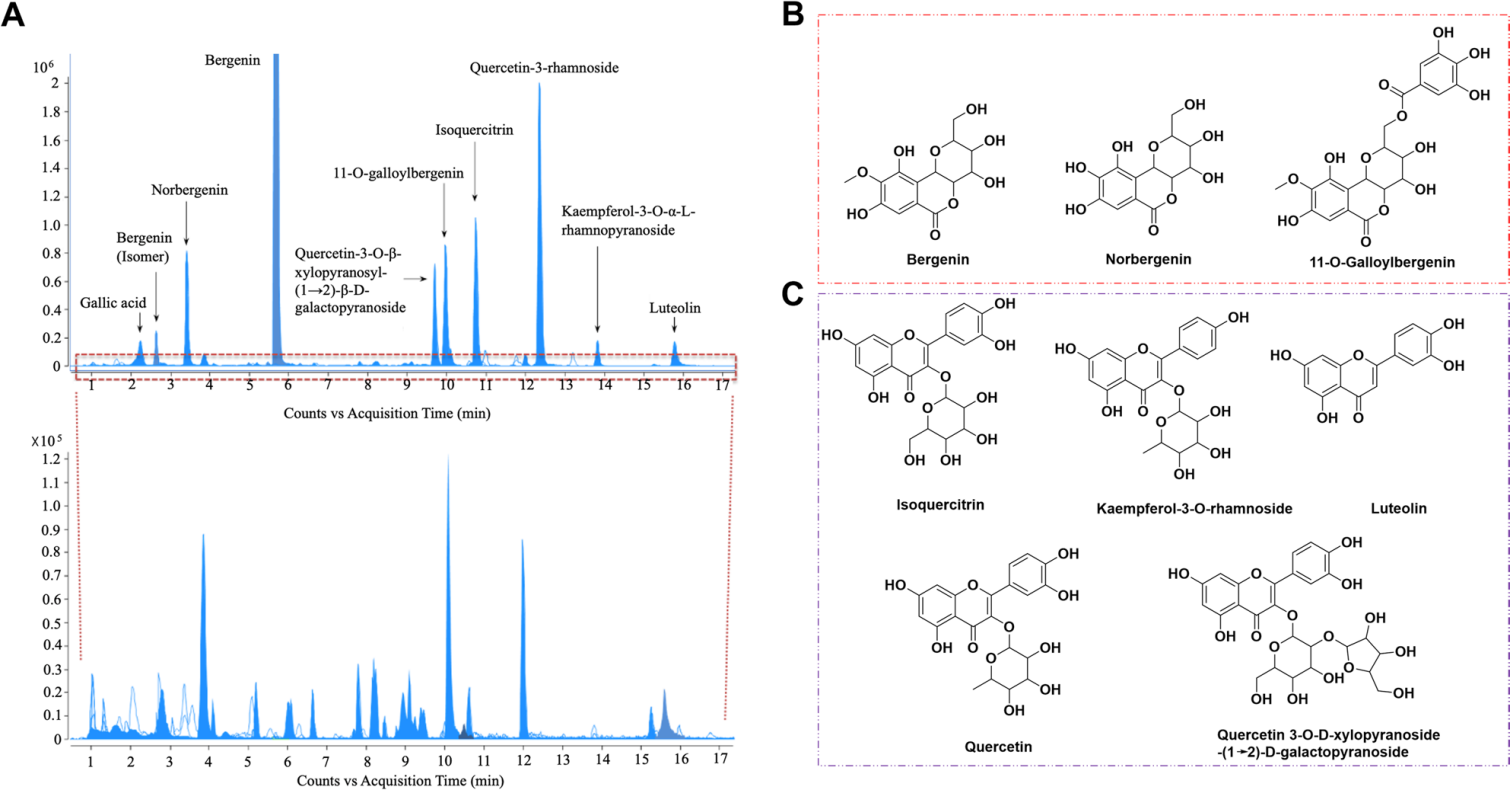
UHPLC-Q-TOF Chemical markers identified by UHPLC-Q-TOF in SSC. **A** UHPLC-Q-TOF total ion chromatogram of 70% ethanol extract from SSC; **b **Structures of the coumarins chemical markers. **C** Structures of the flavonoids chemical markers

Using the UHPLC-Q-TOF technique, we conducted an in-depth analysis of the 70% ethanol extract from SSC. By combining mass spectrometry information and database comparison, 25 components have been successfully identified as major chemical markers (Table 1 and Fig. S2). These compounds, based on their parent core structures, can be primarily classified into coumarins, flavonoids, phenolic acids, and others. The total ion chromatogram (Fig. 2A) clearly illustrates the distribution of these components.

**Table 1:** UHPLC-Q-TOF identification of the chemical markers in SSC

No.	Identification	t_*R*_/min	Formula	Observed m/z	Calculated m/z	MS/MS Fragments
1	α-Hydroxymaltol	1.639	C_6_H_6_O_4_	143.0339	143.0339	100.9671, 116.1060, 127.0395, 107.4457, 136.9318
2	Gallic acid	2.236	C_7_H_6_O_5_	171.0287	171.0288	153.0187, 127.0391, 125.0235, 109.0286, 107.0124, 131.1179, 135.0070
3	Bergenin; 4α,10β-Didehydro	2.799	C_14_H_14_O_9_	327.0712	327.0711	265.1284, 209.0456, 147.0459, 311.1334
4	Norbergenin	3.413	C_13_H_14_O_9_	315.0715	315.0711	195.0295, 237.0393, 249.0395, 261.0398, 279.0504, 297.0606, 267.0498, 233.0452, 219.0291, 207.0290, 191.0346, 177.0180, 167.0344, 103.0386
5	Protocatechuic acid	3.860	C_7_H_6_O_4_	155.0338	155.0339	116.9762, 111.0441, 137.0238, 109.0280
6	Bergenin	5.667	C_14_H_16_O_9_	329.0866	329.0867	311.0764, 263.0555, 209.0451, 251.0558, 275.0556, 293.0663
7	Methyl gallate	6.015	C_8_H_8_O_5_	185.0445	185.0444	153.0183,126.0312
8	4-O-[3,4,5-Trihydroxybenzoyl-(1→6)-β-D-hexopyranosyl]-4-(3,4-Dihydroxyphenyl)-2-butanone	7.689	C_23_H_26_O_12_	495.1491	495.1497	420.0103, 153.0194, 201.0312, 333.0598, 369.6953, 123.0797, 165.0552, 333.0598, 369.6953, 463.0677, 251.1438, 287.0531
9	Thellungianin G	8.153	C_15_H_20_O_4_	265.1436	265.1434	247.1341, 229.1219, 203.1074, 173.0971, 165.0908, 137.0956, 123.0801, 55.0553
10	(E)-1,5-Tridecadiene-7,9-diyn-4-ol	8.253	C_13_H_16_O	189.1271	189.1274	131.0859, 171.1174, 109.1014, 177.1323, 133.1021, 159.1156
11	Benzyl-O-α-L-rhamnopyranosyl-(1→6)-β-D-glucopyranoside	8.468	C_19_H_28_O_10_	417.1751	417.1755	147.0665, 85.0289, 129.0557, 309.1172, 163.0605, 217.0816, 257.0473, 295.1015
12	(7’R,8’R)-4,7’-Epoxy-3’-methoxy-4’,5,9,9’-lignanetetrol-9’-hexoside	8.783	C_25_H_32_O_11_	509.2009	509.2017	329.1422, 341.0659, 455.2353, 477.0634, 317.1407, 107.0610, 163.0737, 299.9609, 492.2553
13	p-Hydroxycinnamic acid	8.949	C_9_H_8_O_3_	165.0548	165.0546	147.0439, 119.0488
14	Quercetin-3-O-β-D-xylopyranosyl-(1→2)-β-D-galactoside	9.695	C_26_H_28_O_16_	597.1456	597.1450	303.0507, 465.1031, 163.0602, 579.1340
15	11-O-Galloylbergenin	9.976	C_21_H_20_O_13_	481.0978	481.0977	153.0185, 293.0660, 275.0552, 209.0450, 251.0554, 311.0765, 463.0873,445.0771, 329.0868, 427.0671, 403.0679
16	Quercetin-5-O-β-D-glucoside	10.092	C_21_H_20_O_12_	465.1027	465.1028	303.0503, 145.0492, 127.0411, 123.7580, 420.8933, 348.7459, 449.0694, 255.9920, 335.1010
17	Rutin	10.523	C_27_H_30_O_16_	611.1604	611.1607	465.1042, 303.0511, 129.0549
18	Isoquercitrin	10.739	C_21_H_20_O_12_	465.1031	465.1028	303.0480, 449.2110, 145.0497, 127.0381, 179.0659, 357.1696, 265.1222
19	Hyperoside	10.988	C_21_H_20_O_12_	465.1026	465.1028	303.0505, 145.0492, 127.0387, 153.0193, 109.0279, 447.0896, 411.0755, 229.0496, 163.0607
20	11-O-3,5-Dihydroxy-4-methoxyphenylbutyrate bergenin	11.999	C_22_H_22_O_13_	495.1131	495.1133	167.0349, 209.0446, 227.0560, 251.0553, 275.0549, 293.0653, 311.0752, 366.0406, 477.1006, 459.0899, 439.9830
21	Quercitrin	12.363	C_21_H_20_O_11_	449.1083	449.1078	303.0504, 129.0546, 147.0655, 391.9384, 103.0381, 287.0523
22	Kaempferol-3-O-α-L-rhamnopyranoside	13.822	C_21_H_20_O_10_	433.1131	433.1129	287.0547, 129.0543, 147.0647, 103.0381, 375.9445, 343.1250, 418.1711
23	Kaempferol	15.397	C_15_H_10_O_6_	287.0552	287.0550	153.0193, 135.0443, 241.0504, 269.0453, 117.0350, 161.0248, 179.0356, 213.0541, 259.0598
24	Quercetin	15.526	C_15_H_10_O_7_	303.0501	303.0499	229.0502, 153.0187, 137.0240
25	Luteolin	15.778	C_15_H_10_O_6_	287.0551	287.0550	153.0187, 135.0442, 117.0335, 161.0235, 241.0504, 269.0449, 185.0608, 213.0548

Notably, the top 8 chemical components with the highest response areas are mainly coumarins and flavonoids. The coumarins include bergenin, norbergenin, and 11-O-Galloylbergenin (Fig. 2B), while the main flavonoids include isoquercitrin, kaempferol-3-O-rhamnoside, luteolin, quercetin, and quercetin 3-O-D-xylopyranoside-(1→2)-D-galactopyranoside (Fig. 2C). Bergenin has the highest peak area (the main compound), followed by 11-O-galloylbergenin and quercetin. It makes the chemical composition of SSC more comprehensive. A total of 25 compounds were identified, among which α-Hydroxymaltol, 4-O-[3,4,5-trihydroxybenzoyl-(1→6)-hexopyranosyl]-4-(3,4-dihydroxyphenyl)-2-butanone, thellungianin G, (E)-1,5-tridecadiene-7,9-diyn-4-ol, (7′R,8′R)-4,7′-epoxy-3′-methoxy-4′,5,9,9′-lignanetetrol-9′-hexoside, 11-O-3,5-Dihydroxy-4-methoxyphenylbutyrate bergenin, methyl gallate, 11-O-galloylbergenin, rutin, hyperoside, and luteolin are tentatively identified for the first time from the Chinese herbal medicine SSC [24–27].

### Construction of SSC, disease targets, and signal pathway networks

**Fig. 3:**
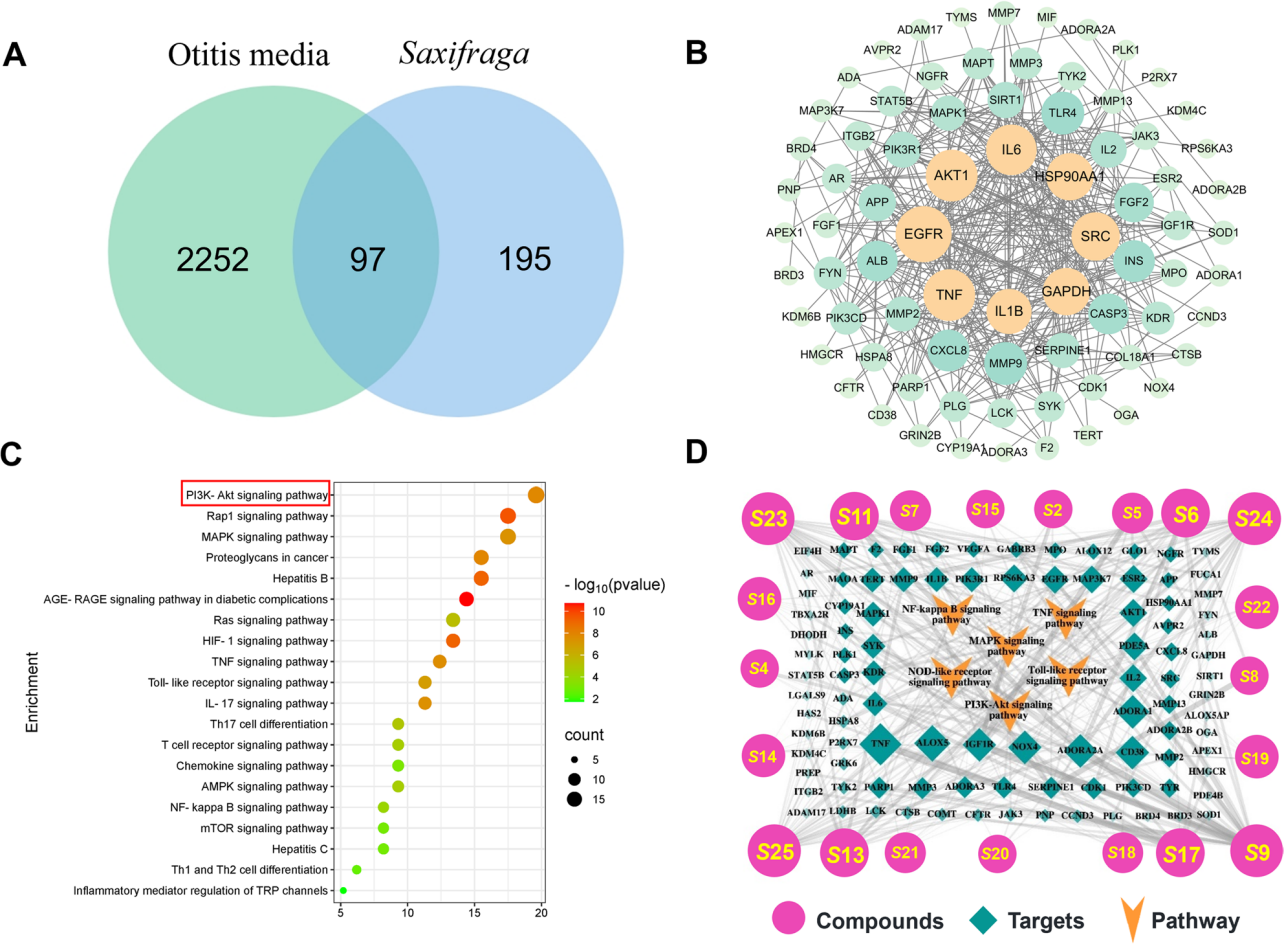
Network pharmacology analysis of active compounds in SSC for the treatment of OM. **A** Venn diagram of SSC targets and OM targets; **b** PPI network of related targets; **c** Bubble chart of KEGG-enriched pathways; **d** Network diagram of active components, disease targets, and signaling pathways in SSC for OM

Using the SwissTargetPrediction database (http://swisstargetprediction.ch/) to predict targets for 25 active compounds identified from SSC using UHPLC-Q-TOF technology. After removing duplicates, a total of 292 targets were obtained. By integrating disease target information from the Genecards database (https://www.genecards.org/) and the OMIM database (https://www.omim.org/), we identified 2649 OM-related disease targets. When these targets were imported into the online bioinformatics drawing tool platform (https://www.bioinformatics.com.cn/), a Venny diagram was created, revealing 97 overlapping targets (Fig. 3A).

Using the STRING database and Cytoscape 3.8.2, we constructed a protein-protein interaction (PPI) network map for the treatment of OM with SSC (Fig. 3B). In the diagram, “nodes” represent targets, and the larger the node’s area, the more connections it has with other nodes. Key hub targets include Tumor necrosis factor (TNF), Glyceraldehyde-3-phosphate dehydrogenase (GAPDH), Protein Kinase B alpha (AKT1), Interleukin-6 (IL-6), Epidermal growth factor receptor (EGFR), Interleukin-1β (IL-1β), SRC proto-oncogene, non-receptor tyrosine kinase (SRC), and Heat shock protein 90 alpha family class A member 1 (HSP90AA1). These targets have close associations with other targets in the network, suggesting they likely play crucial roles in the treatment of OM with SSC.

KEGG enrichment analysis yielded a total of 141 signaling pathways. After filtering and ranking, the top 20 pathways are shown in Fig. 3C. The analysis revealed that the 97 common targets and disease-overlapping targets are mainly enriched in inflammation-related pathways such as the PI3K-Akt, Rap1, TNF, Toll-like receptor, and NF-κB signaling pathways. This indicates that SSC exerts anti-inflammatory effects by targeting these multiple signaling pathways, thereby achieving therapeutic effects for OM.

Matching the active compounds of SSC with OM disease targets, we retained data with matching results and deleted those without. Through KEGG inflammation signaling pathway screening, we obtained a database including 21 active compounds, 97 disease targets, and 6 key pathways. Using Cytoscape 3.8.2 software, we constructed a “active compounds-disease targets-signal pathways” network map for the treatment of OM with SSC and analyzed the network’s topological parameters with Cytoscape’s built-in Network Analyzer plugin. As shown in Fig. 3D, the network contains 124 nodes and 333 edges. Nodes with darker colors and larger areas indicate higher importance within the network; edges with darker colors and greater widths indicate stronger connections between the nodes they connect. Targets related to active compounds in the network include Luteolin (degree: 34), Kaempferol (degree: 33), Quercetin (degree: 33), Bergenin (degree: 11), and Quercetin 3-O-β-D-xylopyranosyl-(1→2)-β-D-galactopyranoside (degree: 9). Each active compound corresponds to multiple targets, and each target is connected to multiple compounds. This suggests that SSC exerts therapeutic effects for OM by acting through multiple compounds on multiple targets.

The “active compounds-disease targets-signal pathways” network revealed that active compounds strongly correlated with the enriched signaling pathways mainly include flavonoids such as Kaempferol, Quercetin, Luteolin, and Rutin, as well as coumarin compounds such as Bergenin and 11-Galloylbergenin, and the small molecule compound Thellungianin G. In-depth studies on the anti-inflammatory activity of Luteolin have identified its mechanisms, including: (1) acting as an inducible NO synthase inhibitor, downregulating NO synthase expression and reducing NO production; (2) activating antioxidant enzymes, scavenging reactive oxygen species (ROS) and inhibiting their production; (3) inhibiting the expression of pro-inflammatory factors like leukotrienes; (4) inhibiting the NF-κB pathway, protein kinase B (AKT), and mitogen-activated protein kinase (MAPK) pathways [28–30]. It has been proven that flavonoids such as Kaempferol, Quercetin, Hyperoside, and Rutin possess anti-inflammatory activity. Bergenin can act as an SIRT1 inhibitor, blocking the NF-κB and MAPK signaling pathways to inhibit inflammatory responses [12, 31, 32]. The literature survey results are consistent with the predictions of this study, verifying the accuracy of the research findings. Through the above analysis, we constructed a detailed network map of active compounds from SSC, OM disease targets, and signal pathways, providing important theoretical basis for further understanding the anti-inflammatory mechanisms and potential therapeutic effects of SSC.

### Results of molecular docking

**Fig. 4:**
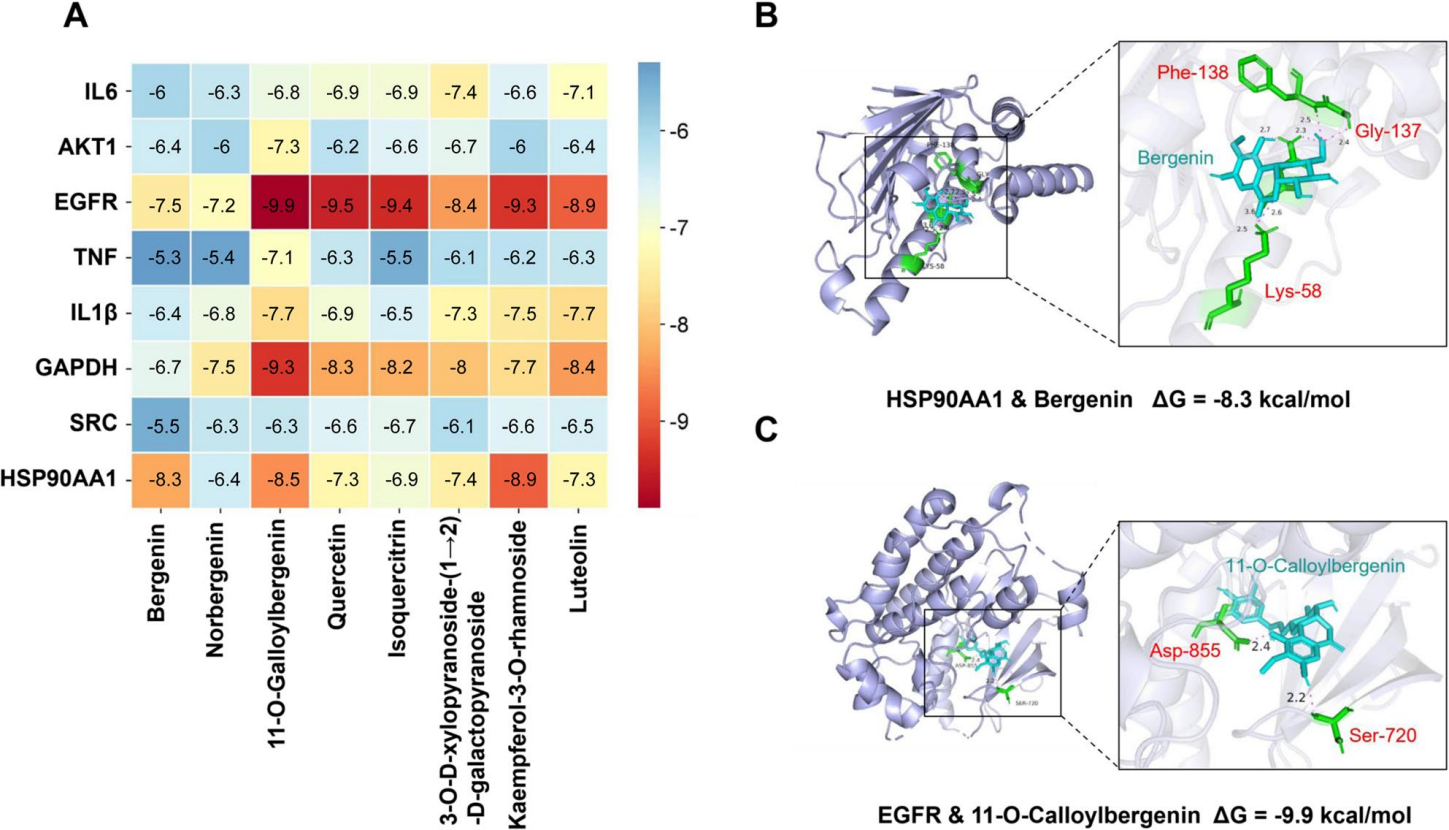
Molecular docking. **A** Matrix diagram of molecular docking between core components and key hub targets/core targets; **b** Visual representation of the docking of HSP90AA1 & Bergenin; **c** Visual representation of the docking of EGFR & 11-O-Calloylbergenin

Validation of the binding between core components and key target proteins or hub targets for treating OM in SSC was performed using the AutoDockTools-1.5.6. ΔG serves as the evaluation parameter for the docking results, reflecting the free binding energy between receptor proteins and ligand molecules, measured in kcal/mol. A negative ΔG value indicates spontaneous binding between the receptor and ligand, particularly when ΔG ≤ -7 kcal/mol, suggesting strong affinity and stable binding between the ligand and receptor [33]. The docking scenario of core components with major hub target proteins is illustrated in Fig. 4A; Tables 2, 3, 4, 5, 6, 7, 8, 9, 10, 11 and 13. Among the 25 components of SSC, bergenin was identified as the most abundant compound based on UHPLC-Q-TOF analysis. It also exhibited favorable binding affinity toward HSP90AA1, with a docking score of ΔG = − 8.3 kcal/mol. Therefore, based on the dual criteria of high abundance and acceptable binding energy, bergenin was prioritized for subsequent experimental validation.

Further visualization of the active pocket of bergenin with HSP90AA1 is presented by PyMOL in Fig. 4B, revealing hydrogen bonding between bergenin and amino acids Lys-58, Gly-137, and Phe-138 of HSP90AA1. HSP90AA1 functions as a molecular chaperone, modulating substrate recognition, ATPase cycling, and chaperone activities through dynamic interactions with co-chaperones. Notably, HSP90AA1 also binds to bacterial lipopolysaccharide (LPS), mediating LPS-induced inflammatory responses involving monocyte secretion of TNF, underscoring the crucial link between this docking result and inflammatory pathways [34, 35].

Further investigation indicates that EGFR exhibits favorable binding energies with most core components, with optimal binding achieved alongside 11-O-Galloylbergenin, yielding ΔG = -9.9 kcal/mol. Visualization of the active pocket of 11-O-Galloylbergenin with EGFR (Fig. 4C) reveals hydrogen bonding with Ser-720 and Asp-855 of EGFR. EGFR is typically expressed on the surface of normal epithelial cells but often overexpressed in certain cancer cells, contributing to metastasis, invasion, and poor prognosis. The downstream signaling pathways of EGFR include the Ras/Raf/MEK/ERK-MAPK and PI3K/Akt/mTOR pathways, which are closely related to inflammation, reinforcing the significance of this docking result in the context of inflammatory pathways [36, 37].

### Effects of SSC on the mRNA expression of IL-6, TNF-α, and IL-1β

The impact of SSC at various concentrations on the viability of J774A.1 cells was assessed using the CCK-8 assay. As illustrated in Fig. S3, at dosages of 6.25, 12.5, 25, 50, and 100 µg/ml, the cell survival rates showed no significant differences compared to the control group (Con), indicating minimal impact of these concentrations on the cells. However, when dosages were elevated to 200 and 400 µg/ml, significant reductions in cell viability were observed, suggesting substantial cytotoxicity of these high concentrations to J774A.1 cells, possibly impairing their normal growth. Consequently, to prevent potential interference with subsequent experiments, these high concentrations were not employed in our study.

**Fig. 5:**
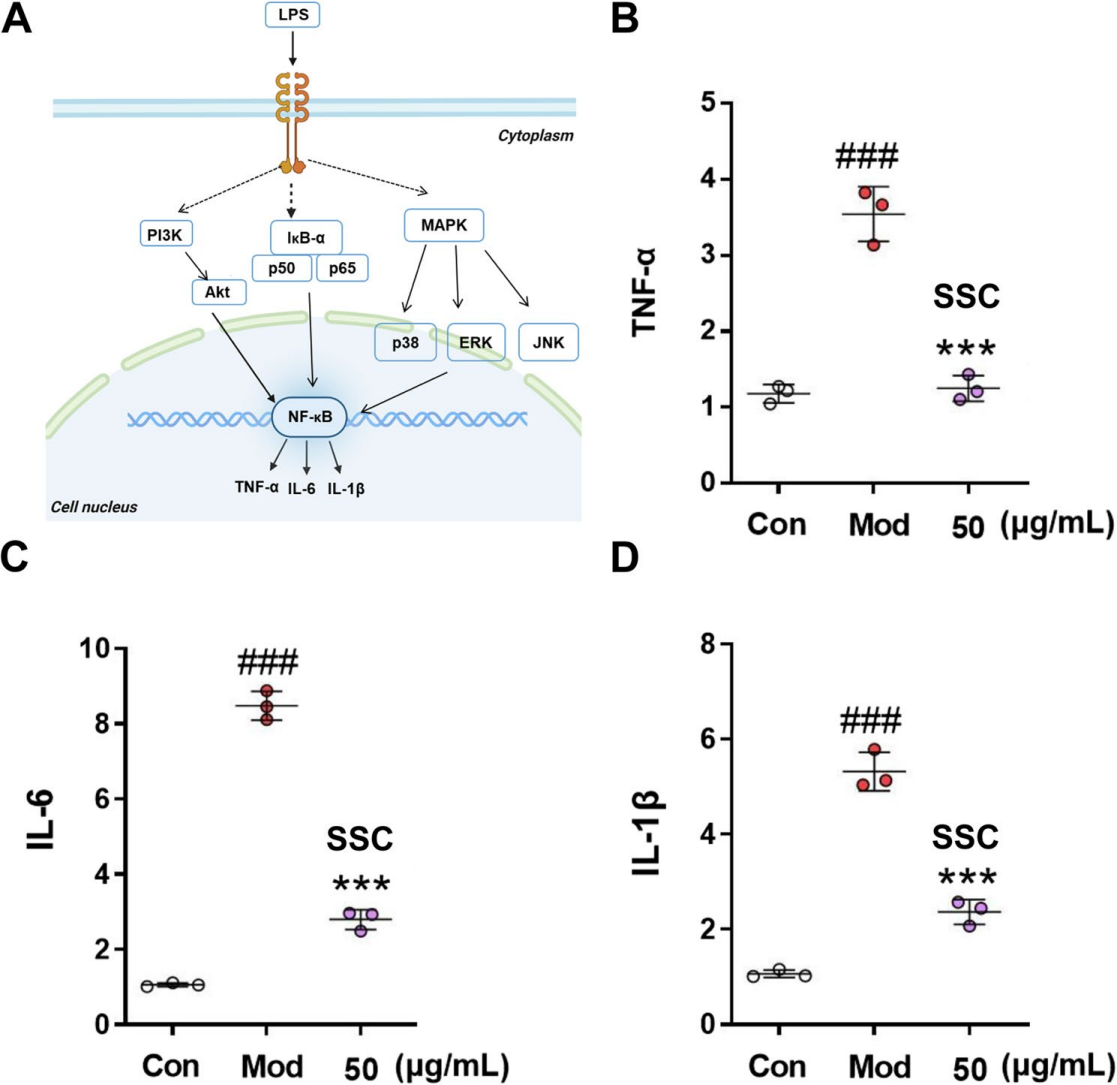
Effects of SSC on the mRNA expression in LPS-induced J774A.1 cells. **A** The action mode diagram of LPS stimulation in generating inflammatory factors. **B***TNF-α*. **C***IL-6*. **D***IL-1β* (*n* = 3, ^###^*P <* 0.001 vs. Con, ***P < 0.001 vs. Mod)

Inflammatory signaling can be induced by LPS, with Toll-like receptor 4 (TLR4) serving as its primary recognition receptor [38]. Upon binding to TLR4, LPS activates this receptor, which subsequently propagates signals *via* Myeloid differentiation primary response gene 88 (MyD88). The interaction between MyD88 and TLR4 triggers downstream signaling cascades involving multiple kinases and transcription factors. In this process, NF-κB is released from the cytoplasm and translocates to the nucleus, initiating the transcription of pro-inflammatory genes [39]. The detailed action mechanism is depicted in Fig. 5A.

The effects of SSC on the mRNA expression of *IL-6*, *TNF-α*, and *IL-1β* in LPS-induced J774A.1 cells are presented in Fig. 5B-D. Compared to the model group, treatment with SSC at doses of 50 µg/ml resulted in significant reductions in the mRNA levels of *IL-6*, *TNF-α*, and *IL-1β*. In vitro experimental results reveal that under LPS induction, SSC notably decreases the mRNA expression levels of *IL-6*, *TNF-α*, and *IL-1β* in J774A.1 cells, thereby exhibiting anti-inflammatory properties [40, 41]. This finding further corroborates the conclusions from previous network pharmacology studies.

## Conclusion

This study elucidated the chemical basis, antibacterial activity, and molecular mechanisms underlying SSC’s efficacy against OM using an integrative research approach. Phytochemical analysis identified key bioactive components in SSC, while antibacterial assays revealed that SSC total extract exhibited broader and stronger activity against OM-associated bacteria than its total flavonoid fraction, suggesting non-flavonoid constituents contribute to its antibacterial effects.

In this study, SSC displayed selective broad-spectrum antibacterial activity, whose efficacy is more pronounced when compared with other plant extracts reported in the literature. Although some studies have indicated that the ethanol extract of *Curcuma longa* (turmeric) at a high concentration (200 mg/mL) shows a slightly larger inhibition zone (18.56 ± 0.46 mm) against *Staphylococcus aureus* than SSC [42], SSC’s inhibitory effects on clinically common KP and PA are significantly superior to those of ethanol extracts of *Zingiber officinale* (ginger) and *Allium sativum* (garlic) reported in the literature [43]. This difference in antibacterial activity may stem from innate differences in the action targets and sensitivity of characteristic bioactive components from different plants to specific pathogenic bacteria.

Additionally, network pharmacology and in vitro experiments confirmed SSC’s anti-inflammatory activity, which is mediated by modulating key signaling pathways (TNF/MAPK/NF-κB) and downregulating pro-inflammatory cytokines. Our findings revealed that bergenin may effectively suppress both the NF-κB and MAPK pathways by reversing IκB-α degradation, reducing NF-κB p65 phosphorylation, and markedly inhibiting the phosphorylation of ERK, JNK, and p38—suggesting that bergenin plays a pivotal role in SSC’s bioactivity [44, 45]. Shi et al. demonstrated *via* cellular thermal shift assay (CETSA) that quercetin binds to EGFR and inhibits ferroptosis and inflammatory responses [46]. Almatroodi et al. reported that luteolin significantly reduces the levels of multiple cytokines, including IL-6, IFN-β, TNF-α, and IL-1β, and blocks MAPK phosphorylation, IκB degradation, and NF-κB activation [47].

Collectively, these findings demonstrate that SSC exerts anti-OM effects *via* a “dual synergistic mechanism” (antibacterial and anti-inflammatory), validating its traditional use for inflammatory ear disorders and providing a scientific basis for developing SSC-based therapeutics to address limitations of current OM treatments. Due to the probabilistic nature of target prediction in network pharmacology, these computational predictions cannot replace experimental validation per se, and their ultimate accuracy needs to be confirmed through laboratory experiments. The study is limited to in vitro gene expression analysis, which cannot fully reflect the complex in vivo microenvironment. Thus, further in vivo validation is required. We will conduct subsequent experiments in preclinical rodent models to verify our findings and improve the translational significance of this work.

Further validation at the animal level is still required. Although EGFR, AKT1 and NF-κB pathway proteins were predicted as potential targets of bergenin by computational analysis, they were not experimentally validated in this study. The main aim of this work was to assess the anti-inflammatory efficacy of SSC. Whether bergenin directly regulates these targets will be verified by SPR, CETSA and gene knockdown experiments in our ongoing research. Nevertheless, SSC significantly inhibited IL-6, TNF-α and IL-1β at both mRNA and protein levels, supporting its anti-inflammatory activity. Additionally, LC-MS analysis results have not yet clarified the specific contents of individual components through quantitative analysis, nor have they established strict dose-effect relationships between the concentrations of specific components and the observed biological effects. Future studies will focus on isolating non-flavonoid antibacterial components from SSC and verifying their in vivo efficacy in OM animal models. Based on traditional otic drop formulations, SSC total extract will be developed into an otic gel to assess its efficacy in reducing middle ear inflammation and clearing pathogenic bacteria in a rat OM model. These efforts aim to validate SSC’s therapeutic potential and promote its clinical application for OM.

## Supplementary Information

Supplementary Material 1.

## Data Availability

Data is provided within the manuscript or supplementary information files.

## Abbreviations

OM: Otitis media
UHPLC-Q-TOF-MS: Ultra-high performance liquid chromatography-quadrupole time-of-flight mass spectrometry
SSC: Saxifraga stolonifera curtis
AKT1: AKT serine/threonine kinase 1
EGFR: Epidermal growth factor receptor
KEGG: Kyoto encyclopedia of genes and genomes
LPS: Lipopolysaccharide
MAPK: Mitogen-activated protein kinase
NF-κB: Nuclear factor-kappa B
PPI: Protein-protein interaction
qPCR: Quantitative polymerase chain reaction
SRC: SRC proto-oncogene, non-receptor tyrosine kinase
TNF: Tumor necrosis factor
IL: Interleukin
