# Supplementary material for: Saxifraga stolonifera curtis for ear inflammation: antibacterial efficacy and key bioactive constituents
Source: BMC Complement Med Ther. 2026 Apr 13;26:191. doi: 10.1186/s12906-026-05346-x (PMC13188436; doi:10.1186/s12906-026-05346-x)
Supplement: Supplementary file 1 — Supplementary Material 1. [file 12906_2026_5346_MOESM1_ESM.docx]

**Supporting Information**

***Saxifraga stolonifera* Curtis for Ear Inflammation: Antibacterial Efficacy and Key Bioactive Constituents**

Fukui Shen ^1,†^, Hongyu Liu ^1,†^, Wen Yang ^2,†^, Tian-Tian Tong ^3^, Shiyu Guo ^1^, Zhihao Wei ^1^, Wenping Zhang ^1^, Jihui Fan ^1^, Yuqing Zhang ^1,*^ and Hao Huang ^1,^[[1]](#footnote-1)^*^

*1 Jiangxi Province Key Laboratory of Pharmacology of Traditional Chinese Medicine, School of Pharmacy, Gannan Medical University, Ganzhou 341000, China.*

*2. State Key Laboratory of Medicinal Chemical Biology, College of Pharmacy and Tianjin Key Laboratory of Molecular Drug Research, Nankai University, Tianjin 300353, China.*

*3 State Key Laboratory of Quality Research in Chinese Medicine, Macau University of Science and Technology, Avenida Wai Long, Taipa, Macau SAR, China.*

*† These authors contributed equally to this study.*

**Table S1** Primer Sequences.

| Primer Name |  | Sequences |
| --- | --- | --- |
| GAPDH | forward 5′-3′ | GGAGAGTGTTTCCTCGTCCC |
|  | reverse 3′-5′ | GATGGGCTTCCCGTTGATGA |
| IL-6 | forward 5′-3′ | CTGCAAGAGACTTCCATCCAG |
|  | reverse 3′-5′ | AGTGGTATAGACAGGTCTGTTGG |
| TNF-α | forward 5′-3′ | CGGGCAGGTCTACTTTGGAG |
|  | reverse 3′-5′ | ACCCTGAGCCATAATCCCCT |
| IL-1β | forward 5′-3′ | TGAAGTTGACGGACCCCAAA |
|  | reverse 3′-5′ | CAGCCACAATGAGTGATACTGCC |


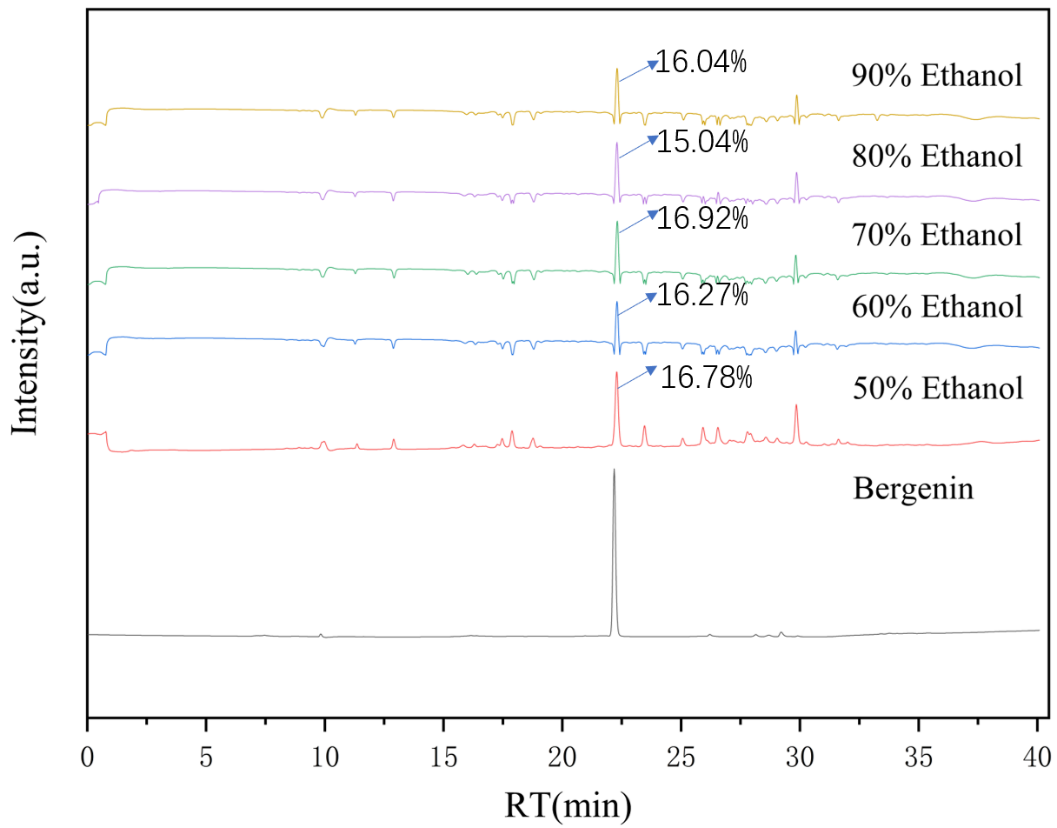


**Fig. S1** HPLC analysis of extraction process optimization with bergenin as the reference

**Fig. S2** 25 The structures of the major compounds contained in SSC


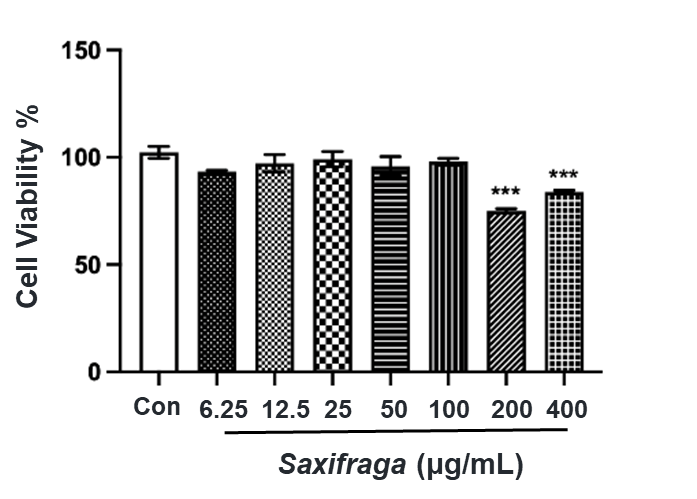


**Fig. S3** Toxicity of SSC to J774A.1 cells (*n* = 6), *** *P*< 0.001 *vs.* Con.

**Table S2** Bergenin-HSP90AA1

| No. | affinity (kcal/mol) | RMSD （Å） |
| --- | --- | --- |
| 1 | -8.3 | 0.000 |
| 2 | -6.2 | 1.340 |
| 3 | -5.8 | 1.549 |
| 4 | -5.8 | 2.212 |
| 5 | -5.7 | 2.037 |
| 6 | -5.7 | 2.145 |
| 7 | -5.7 | 1.892 |
| 8 | -5.7 | 2.031 |
| 9 | -5.6 | 2.154 |

**Table S3** Norbergenin-HSP90AA1

| No. | affinity (kcal/mol) | RMSD （Å） |
| --- | --- | --- |
| 1 | -6.4 | 0.000 |
| 2 | -6.3 | 2.968 |
| 3 | -6.1 | 2.065 |
| 4 | -6.0 | 1.854 |
| 5 | -5.9 | 1.639 |
| 6 | -5.9 | 2.361 |
| 7 | -5.8 | 2.546 |
| 8 | -5.6 | 2.894 |
| 9 | -5.6 | 2.624 |

**Table S4** Quercitrin-HSP90AA1

| No. | affinity (kcal/mol) | RMSD （Å） |
| --- | --- | --- |
| 1 | -7.3 | 0.000 |
| 2 | -6.9 | 6.115 |
| 3 | -6.8 | 3.774 |
| 4 | -6.8 | 3.494 |
| 5 | -6.8 | 2.494 |
| 6 | -6.7 | 9.128 |
| 7 | -6.7 | 9.082 |
| 8 | -6.5 | 4.257 |
| 9 | -6.5 | 3.084 |

**Table S5** Luteolin- HSP90AA1

| No. | affinity (kcal/mol) | RMSD （Å） |
| --- | --- | --- |
| 1 | -7.3 | 0.000 |
| 2 | -6.5 | 13.119 |
| 3 | -6.5 | 19.944 |
| 4 | -6.4 | 20.035 |
| 5 | -6.4 | 20.912 |
| 6 | -6.3 | 20.402 |
| 7 | -6.1 | 19.700 |
| 8 | -6.1 | 11.656 |
| 9 | -6.1 | 17.683 |

**Table S6** Bergenin-EGFR

| No. | affinity (kcal/mol) | RMSD （Å） |
| --- | --- | --- |
| 1 | -7.5 | 0.000 |
| 2 | -7.2 | 5.439 |
| 3 | -7.1 | 4.373 |
| 4 | -7.1 | 1.694 |
| 5 | -6.6 | 18.001 |
| 6 | -6.5 | 2.841 |
| 7 | -6.5 | 4.127 |
| 8 | -6.4 | 4.528 |
| 9 | -6.4 | 10.005 |

**Table S7** 11-O-Calloylbergenin-EGFR

| No. | affinity (kcal/mol) | RMSD （Å） |
| --- | --- | --- |
| 1 | -9.9 | 0.000 |
| 2 | -9.2 | 1.429 |
| 3 | -9.2 | 1.732 |
| 4 | -9.0 | 1.694 |
| 5 | -9.0 | 2.01 |
| 6 | -8.4 | 2.51 |
| 7 | -8.4 | 1.952 |
| 8 | -8.3 | 1528 |
| 9 | -8.3 | 1.005 |

**Table S8** Quercitrin -EGFR

| No. | affinity (kcal/mol) | RMSD （Å） |
| --- | --- | --- |
| 1 | -9.5 | 0.000 |
| 2 | -8.9 | 2.646 |
| 3 | -8.8 | 1.785 |
| 4 | -7.7 | 3.059 |
| 5 | -7.7 | 3.656 |
| 6 | -7.6 | 15.765 |
| 7 | -7.3 | 15.328 |
| 8 | -7.2 | 16.630 |
| 9 | -7.0 | 3.776 |

**Table S9** Luteolin- EGFR

| No. | affinity (kcal/mol) | RMSD （Å） |
| --- | --- | --- |
| 1 | -8.9 | 0.000 |
| 2 | -8.8 | 1.480 |
| 3 | -7.9 | 2.190 |
| 4 | -7.7 | 3.363 |
| 5 | -7.7 | 2.485 |
| 6 | -7.7 | 2.440 |
| 7 | -7.5 | 3.365 |
| 8 | -7.4 | 3.473 |
| 9 | -7.3 | 2.971 |

**Table S10** Bergenin -SRC

| No. | affinity (kcal/mol) | RMSD （Å） |
| --- | --- | --- |
| 1 | -5.5 | 0.000 |
| 2 | -5.5 | 1.540 |
| 3 | -5.4 | 1.744 |
| 4 | -5.3 | 12.844 |
| 5 | -5.3 | 2.026 |
| 6 | -5.3 | 1.385 |
| 7 | -5.1 | 18.762 |
| 8 | -5.1 | 2.030 |
| 9 | -5.1 | 1.862 |

**Table S11** 11-O-Calloylbergenin-SRC

| No. | affinity (kcal/mol) | RMSD （Å） |
| --- | --- | --- |
| 1 | -6.3 | 0.000 |
| 2 | -6.3 | 15.912 |
| 3 | -6.3 | 15.596 |
| 4 | -6.2 | 15.856 |
| 5 | -6.1 | 2.152 |
| 6 | -6.0 | 1.930 |
| 7 | -6.0 | 2.351 |
| 8 | -6.0 | 15.653 |
| 9 | -6.0 | 23.063 |

**Table S12** Quercitrin -SRC

| No. | affinity (kcal/mol) | RMSD （Å） |
| --- | --- | --- |
| 1 | -6.3 | 0.000 |
| 2 | -6.3 | 15.912 |
| 3 | -6.3 | 15.596 |
| 4 | -6.2 | 15.856 |
| 5 | -6.1 | 2.152 |
| 6 | -6.0 | 1.930 |
| 7 | -6.0 | 2.351 |
| 8 | -6.0 | 15.653 |
| 9 | -6.0 | 23.063 |

**Table S13** Luteolin-SRC

| No. | affinity (kcal/mol) | RMSD （Å） |
| --- | --- | --- |
| 1 | -6.5 | 0.000 |
| 2 | -6.4 | 1.199 |
| 3 | -6.4 | 24.145 |
| 4 | -6.3 | 10.200 |
| 5 | -6.3 | 23.404 |
| 6 | -6.3 | 1.677 |
| 7 | -6.2 | 23.504 |
| 8 | -6.2 | 24.522 |
| 9 | -6.0 | 22.519 |


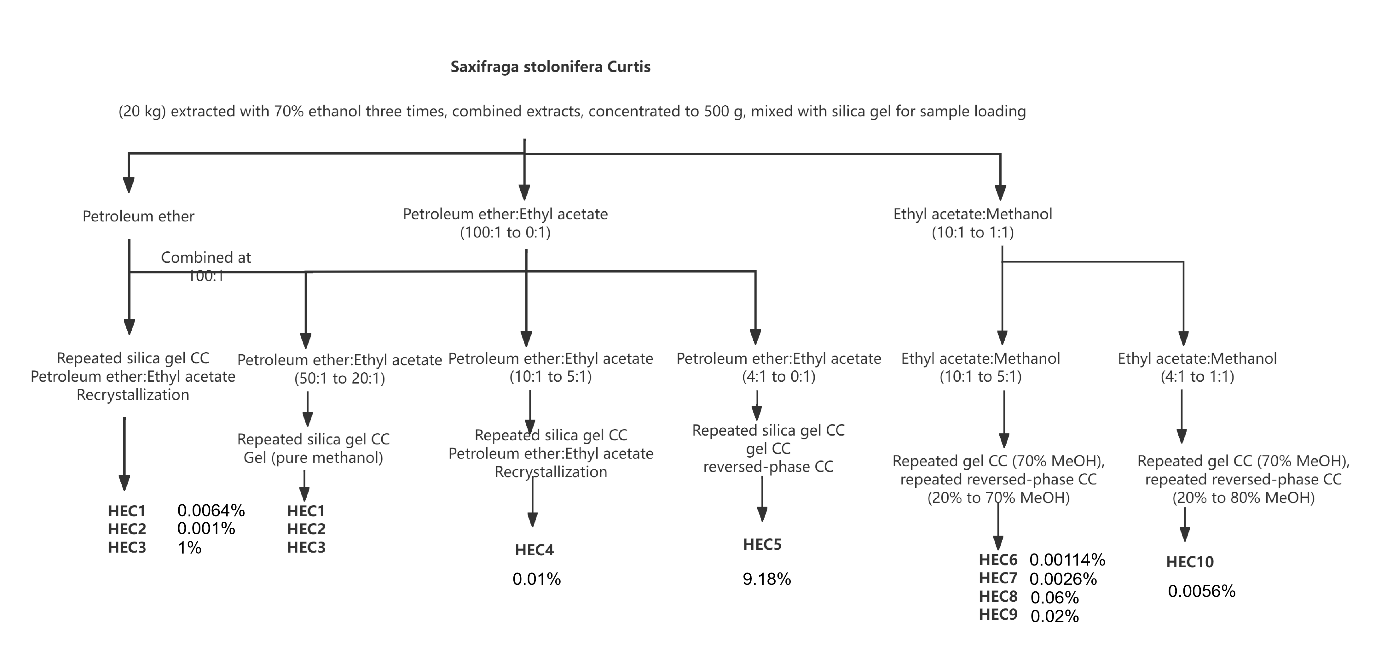


**Fig. S4** Yield diagram of the SSC separation process

1. * Corresponding authors.

   *E-mail addresses*: yqzhang@gmu.edu.cn (Y. Zhang), hhuang@gmu.edu.cn (H. Huang). [↑](#footnote-ref-1)
